# The association between nuclear receptors and ocular diseases

**DOI:** 10.18632/oncotarget.15178

**Published:** 2017-02-07

**Authors:** Ke Liu, Chang Zou, Bo Qin

**Affiliations:** ^1^ Jinan University, Guangzhou, China; ^2^ Shenzhen Eye Hospital, Affiliated Shenzhen Eye Hospital of Jinan University, Joint College of Optometry of Shenzhen University, Shenzhen Key Laboratory of Ophthalmology, Ocular Trauma Treatment and Stem Cell Differentiation Public Service Platform of Shenzhen, Shenzhen, China; ^3^ Clinical Medical Research Center, The Second Clinical Medical College, Shenzhen People's Hospital, Jinan University, Shenzhen, China

**Keywords:** nuclear receptor, ocular disease

## Abstract

Nuclear hormone receptors (NRs) are one of the most abundant transcription factors in the human cells. They regulate expression of genes via interactions with corresponding ligands, co-activators, and co-repressors. These molecular pathways play important roles in the development, cell differentiation, and physiologic and metabolic processes. Increasingly, targeting nuclear receptors is becoming a promising strategy for new drug development. The aim of this review is to discuss the association between nuclear receptors and eye development, and expand their role in various ocular diseases such as keratitis, cataract, glaucoma, uveitis, retinopathy, and ophthalmic tumors. Recent studies in this area are highlighted as well as future research directions and potential clinical applications. Finally, various strategies will be elucidated to inspire more targeted therapies for ocular diseases through the use of nuclear receptors.

## INTRODUCTION

Nuclear receptors (NR) or nuclear hormone receptors are a family of receptors that interact with hormonal factors in nucleus and regulate gene expression. The spectrum of functions they affect is vast and includes development, reproduction, differentiation, metabolism, and homeostasis [1]. The basic signaling pathway of NRs involves a signal production (whether it be endocrine, paracrine, or autocrine), its transport to peripheral organs, ligand binding to the receptor, and transcriptional activation. The ligands commonly consist of lipophilic hormones, such as steroids and retinoids, that diffuse through the lipid bilayer, allowing more flexible physiological activity both intracellularly and extracelluarly [1]. NRs can also sense xenobiotics and play a significant role in detoxification [1]. Finally, there are “orphan receptors” whose ligands do not exist or are yet to be found. Discovering novel ligands and the potential functions for these orphan receptors is an additional path for drug development.

As an evolutionarily conserved yet functionally diverse group of transcription factors, NRs interact directly with DNA response elements of target genes, as well as “cross-talk” with other signaling pathways. The intermediary factors in these pathways, known as co-repressors or co-activators, further modulate transcription. Understanding the orchestrated activity of co-repressor and co-activator complexes has elucidated the general molecular mechanism of various receptor responses, such as those involved in the development and maintenance of photoreceptor cells with rods and cones [7]. NRs control intricate regulatory signaling pathways in human health and disease progression [57]. Particularly, as the focus of this review, NRs are critical in both eye development and pathogenesis of ocular diseases [4-7].

Because of the immense involvement in numerous functions and their affinity to small molecule ligands, NRs have become a strategic therapeutic target for many diseases. With small molecules, drug design can be easily manipulated, and the effect they can have might exceed that of the endogenous ligand counterparts. For example, dexamethasone, a common anti-inflammatory medication, binds to the glucocorticoid receptor (GR) and exerts a higher biological response than cortisol [1]. Ligands for various NRs such as the estrogen receptor (ER) and peroxisome proliferator-activated receptor (PPAR) have been synthetized for pharmacological treatments of cancer and diabetes. Due to the success of these NR-targeted clinical applications, identifying novel signaling pathways and designing agonist/antagonist ligands for NRs have resulted in some promising research initiatives in the area of ophthalmology. The aim of this review is to discuss the basic concepts behind nuclear receptors, the association between nuclear receptors and development of ocular diseases, and highlight recent studies in this area as well as future research directions.

## FAMILY MEMBERS OF NRs SUPERFAMILY

Currently, there are more than 300 hundred members in NRs superfamily across species; and in human, 48 types of NRs are known [1]. There are several nomenclatures for NRs. Initially the NRs were subdivided into steroid hormone receptors and non-steroid hormone receptors based on the ligands being steroidal or non-steroidal. The steroidal hormone receptors include mineralocorticoid receptor (MR), glucocorticoid receptor (GR), androgen receptor (AR), progesterone receptor (PR) and estrogen receptor (ER). The non-steroidal hormone receptors include thyroid hormone receptor (TR), vitamin D3 receptor (VDR), retinoic acid receptor (RAR), retinoid X receptor (RXR) and others. These two types of NRs were subsequently named Type I and Type II NRs respectively. With the development of molecular cloning technology, more and more proteins were found to share a similar structure with the NR superfamily, but no endogenous ligand existed for them or no endogenous ligand was found at the time. Therefore, these receptors were defined as “orphan receptors”. One of the examples is the estrogen-related receptor (ERR). Also, NRs have been subdivided into 4 subtypes according to the DNA binding characteristic and dimerization preferences of NRs. In 1999, the Nuclear Receptors Nomenclature Committee categorized NRs into six subfamilies according to sequence homology, and in the convenience of naming newly discovered NRs, the non-typical NRs with only one-conserved domain were classified into subfamily 0.

## STRUCTURE OF NUCLEAR RECEPTORS (NRs)

**Figure 1:**
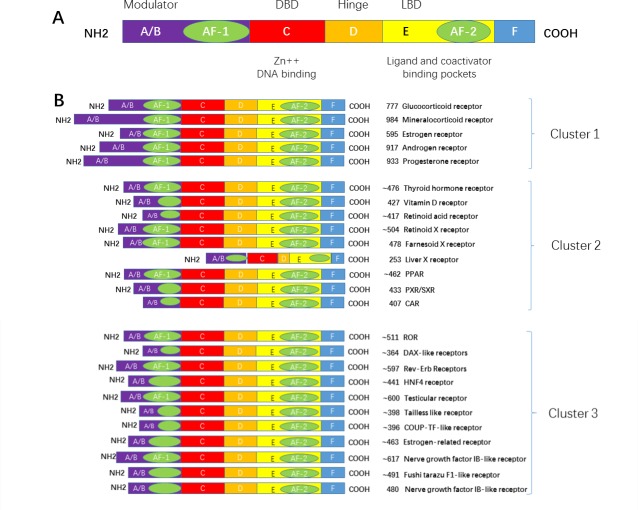
Primary structure of a nuclear receptor A typical nuclear receptor contains a variable N-terminal region **A**./**B**., a conserved DBD **C**., a variable hinge region **D**., a conserved LBD **E**., and a variable C-terminal region **F**. Abbreviations: DBD: DNA binding domain; LBD: ligand binding domain; AF1:activation function 1 region; and AF2: activation function 2 region.

A typical NR contains 5 domains, designated as A/B, C, D, E, and F domain. The A/B domain varied greatly in sequence in different receptors. It contains the activation function 1 (AF-1) which functions independently in binding with a ligand. The C domain is the DNA-binding domain (DBD). It contains two zinc fingers and is a highly conserved domain. The D domain contains the nucleus localization sequence (NLS), and is thought to be a flexible domain that connects the DBD with the E domain, also known as the ligand binding domain (LBD). The largest domain, E domain, has the activation function 2 (AF-2). E domain is highly conserved in structure and moderately conserved in sequence in different nuclear receptors. Its function is not dependent on the ligand. In addition, some NRs contain the F domain, which is localized at the C terminus and varied greatly in sequence in different nuclear receptors; its structure and function remains unclear.(Figure 1)

## REGULATION OF GENE EXPRESSION BY NRs

**Figure 2:**
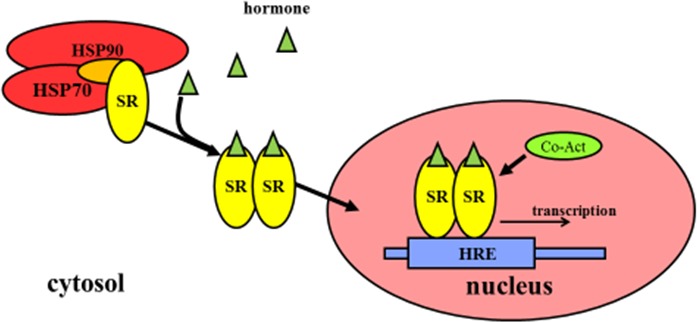
Regulation of gene expression by steroid hormone receptors The inactivated SRs commonly bind to HSPs that prevent the translocation of SRs into the nucleus. Ligand binding to SRs in the cytosol causes the conformational change of SRs, which results in the dissociation of HSPs, translocation from the cytoplasm into the nucleus, binding to HREs as well as recruiting co-activators that are responsible for transcription of downstream DNA into mRNA. Abbreviations: SRs: steroid hormone receptors; HSP: heat shock protein; HREs: hormone response elements; Co-Act: Co-activators.

The inactivated steroid hormone receptors (SRs) commonly bind to heat shock proteins (HSP) such as HSP70 orHSP90 in the cytosol, and HSPs prevents the translocation of SRs into nucleus, thus stopping SRs from binding to DNA in the nucleus. Ligand binding to SRs in the cytosol can induce a conformational change in SRs, which leads to the dissociation of heat shock proteins. The dissociated proteins become homo-dimers and, translocate from the cytosol into the nucleus. Then they bind to the hormone response elements (HREs) that are specific sequences of DNA. They recruit co-activators that regulate the transcription of DNA into mRNA. Mechanistic actions of non-steroid hormone receptors are different from that of SRs (Figure 2).

**Figure 3:**
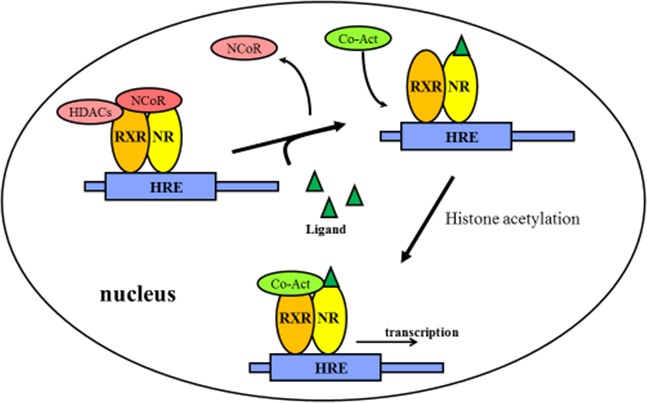
Regulation of gene expression by non-steroidal receptors The non-steroidal receptors are retained in the nucleus regardless of the ligand binding status and bind as heterodimers (usually with RXR) to HREs and complex with co-repressor proteins. Ligand binding to the nuclear receptor causes dissociation of co-repressor and recruitment of co-activator proteins. Abbreviations: NR: non-steroidal receptors; RXR: Retinoid X receptors; HDACs: Histone deacetylases; HREs: hormone response elements; NCoR: nuclear receptor corepressor; Co-Act: Co-activators.

The non-steroid receptors such as TRs, RARs and VDRs, are located in the nucleus. They bind to HREs as heterodimers. They are then complexed to co-repressor proteins, which in turn activate histone deacetylase. The histone deacetylation makes a more condensed conformation of histone proteins near HREs, thus inhibiting target gene transcription. After the ligands bind to the nuclear receptor, the co-repressors are dissociated and co-activator proteins are recruited. RNA polymerase and other additional proteins are also recruited to the NR/DNA complex that initiate the transcription of DNA into mRNA (Figure 3).

## NRs AND EYE DEVELOPMENT

**Figure 4:**
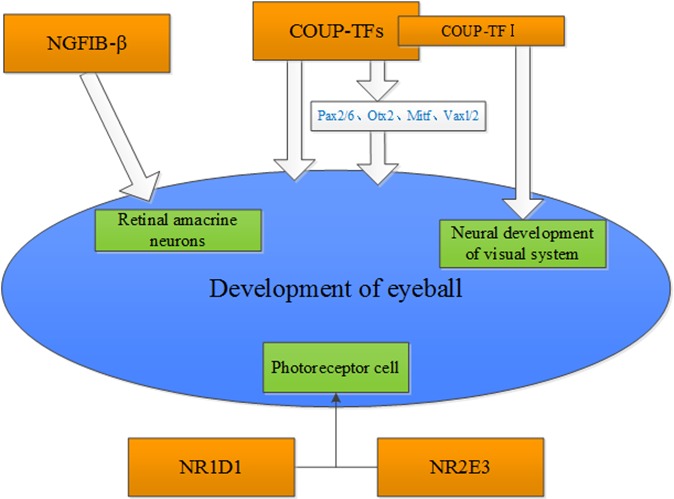
Potential nuclear receptors that are associated with the development of the eye

Olivares AM et al. [2] reviewed the role of nuclear receptors on the eye and brain development and function. They summarized that Vitamin A, RARβ and RA are essential for eye development and maintenance. In addition, Nr2e3 plays a dual regulation role in regulating the development and function of rod and cone cells. Li et al. [3], examined the protein expression of an orphan receptor, NGFIB-β in the rat retina during development by immunohistochemistry. The study showed that NGFIB-β plays a significant role in regulating the differentiation and maturation of rat retinal amacrine neurons. Tang et al. [4] showed that the chicken ovalbumin upstream promoter-transcription factors (COUP-TFs) were highly expressed in the eyes of developing embryos. Pax6 and Otx2 were regulated by COUP-TF1 (also named EAR-3 or NR2FI) and COUP-TFII (also named as ARP-1 and NR2F2), respectively. The analysis on the phenotypic change of COUP-TFI and COUP-TFII conditional single-gene knockout mouse model indicates that COUP-TFI and COUP-TFII compensate for each other to maintain the morphogenesis of the eye. However, progenitor cells at the dorso-distal optic vesicle failed to appropriately differentiate in the eye-specific COUP-TFI/TFII double-gene knockout mouse. Du et al. [5], summarized the role of COUP-TFs in the regulation of the developing eyes in embryo, and found that COUP-TFs regulated the development of embryo eyes via directly or indirectly modulating the eye morphogenesis-related transcriptional factors including Pax2/6, Otx2, Mitf, Vaxl/2 etc. Bosch et al. [6] found that six patients with CVI and/or optic nerve abnormalities, have either deletions of NR2F1(COUP-TFI) or heterozygous missense mutations in NR2F1. Their study suggests that NR2F1 has a significant function in regulating the neurodevelopment of visual system. Disruption of NR2F1 can result in intellectual disability and optic atrophy. Mollema et al. [7] found that Nr1d1 and its cofactor Nr2e3 play an important role in regulating transcriptional networks that are important for the development and function of photoreceptor (Figure 4).

## NRs AND OCULAR DISEASES

### NRs and keratitis

Fungal keratitis (FK) refers to an infective process of the cornea caused by any of the pathological fungi. Most of the fungal keratitis leads to loss of vision or blindness. The treatment of the disease is a challenge because of the lack of effective antifungal drugs that could penetrate into the corneal epithelia. At the beginning of infection, invasion of pathogens are recognized by the immune system and secretion of inflammatory mediators such as cytokines and chemokines may be triggered. The invaded pathogens could be killed by neutrophils recruited by the inflammatory mediators. However, excessive inflammatory mediators need to be reduced in the late stage of disease [8]. All-trans retinoic acid (ATRA) is the carboxylic acid form of vitamin A. ATRA was found to regulate targeted gene expressions to exert its anti-inflammatory and immune modulatory functions via binding to RARα, β, γ and RXR α, β, γ receptors [9]. In addition, ATRA also inhibited the expression of matrix metalloproteinases (MMPs), which play an important role in FK development [10]. Therefore, ATRA has been regarded as a potential treatment modality for FK. Zheng et al. [11], examined the expressions of MMPs in cultured corneal fibroblasts and found that CD347 is a selective agonist of RAP γ. Inhibition of the expression of MMP_1,2,3,9_ can slow down corneal degradation, suggesting a potential role of CD347 in the treatment of corneal ulcer. In a research based on VEGF-induced neovascular rat cornea model, Sarayba et al [12] reported that pioglitazone is effective in decreasing the formation of angiogenesis. In another article, the authors pointed out that rosiglitazone effectively controlled corneal fibrosis *in vivo* and *in vitro*. It provides a basis for the prevention and treatment of corneal scar [13]. It is note worthy that pioglitazone and rosiglitazone are ligands of PPARγ. In the early stage of corneal alkali burn, PPARγ agonists inhibited inflammatory reaction, reduced the fibrotic reaction and prevented the formation of angiogenesis. PPARγ agonists may provide a new method of treatment for keratitis and corneal trauma [14,15].

### NRs and cataract

Various studies have shown that long term use of corticosteroids in patients with autoimmune diseases, allergy and organ transplantation contributes to the development of posterior sub-capsular cataract [16-18]. It was reported that RU486, the glucocorticoid receptor(GR) antagonist, decreased the expression of Na, K-ATPase α1 in the lens of rat. In addition, dexamethasone treatment reduced the expression of vimentin. These results indicate that the decrease of Na, K-ATPase and vimentin mediated by GR may result in the formation of steroid-induced cataract [19, 20]. It was showed that GRs participated in the process of cell apoptosis in human lens. [21]. However, RU486, the glucocorticoid receptor antagonist does not fully rescue the cell apoptosis, which may be due to the failure of RU486 to inhibit Bax expression and its elevation of the caspase-3 protein expression. Colitz et al. [22] found that ERα was up-regulated in epithelial cells of cataractous canine lens, but its underlying mechanisms require further investigation. In addition, cataract associated lens opacification is primarily due to the loss of Ca2^+^ homeostasis and subsequent activation of calpains. It was demonstrated that stimulation of RARα decreased Ca2+ influx into the lens and reduced the over activation of calpains. RARα can potentially be used for prevention and treatment of human diabetic cataracts [23].

### NRs and glaucoma

Glucocorticoids exert biological functions by mediating GR, and have been extensively used as anti-inflammatory drugs and immune suppressants in ocular diseases and for post-ophthalmic surgery. Increasing intraocular pressure is a commonly observed side effect of glucocorticoid treatment. The pathogenesis may be due to the inhibition of the activity of lysosomes by glucocorticoid, which in turn results in an accumulation of mucopolysaccharide due to its reduced clearance by the trabecular meshwork. In addition, the pathogenesis of glucocorticoid-induced glaucoma may be attributed to its modulation of the cytoskeleton trabecular meshwork. The binding between glucocorticoid and GR involves high specificity and saturation, and the effects of glucocorticoid depends on the number of GRs in the cytosol. Therefore, an individual's sensitivity to glucocorticoid depends on the number of GR in the trabecular meshwork. Zhang et al. [24] showed that GR concentration in the peripheral blood lymphocytes of rabbits is proportion to the GR number in the trabecular meshwork. Measurement of GR in the peripheral blood lymphocytes may be helpful in determining the glucocorticoid sensitivity in steroid-induced glaucoma, and may potentially serve as a new prognostic tool. If personalized therapy for glaucoma can be employed based on an individual's sensitivity to glucocorticoids, the incidence of steroid-induced glaucoma maybe significantly reduced. In addition, the antagonist of GR may be a potential new therapy for steroid-induced glaucoma, but the relevant mechanisms will require further investigation. Alternatively, Agapove et al [25], showed that an increased expression of NF-κB and AR in glaucomatous ONH astrocytes suggested that androgen plays an important role in the pathogenesis of glaucoma, but the underlying mechanisms still need to be confirmed.

### NRs and Uveitis

Uveitis is an ocular disease that is most common in adults. It carries a very complicated pathogenesis that can be classified into many types. Most of the uveitis results from the infections and disorders of the immune system. When left untreated, it could progress to blindness. Therefore, uveitis has attracted worldwide attention because of its significant role in the cause of blindness.

**Figure 5:**
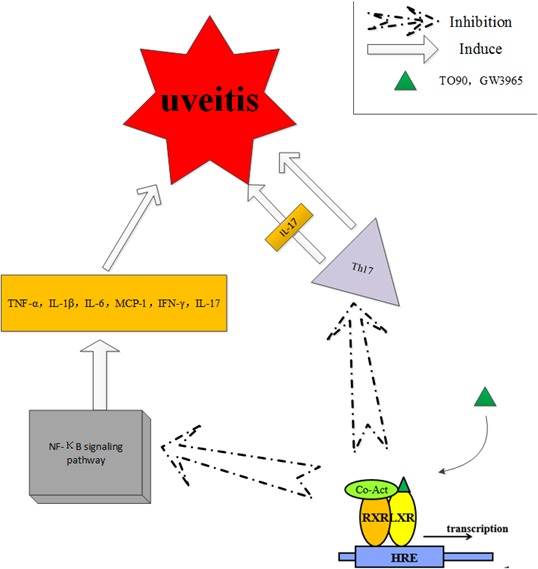
The liver X receptor (LXR) stimulants suppressed the inflammatory response and blocked the signal transduction of NF-κB signaling, which plays an important role in uveitis

The activation of NF-κB signaling induces the expression of inflammation-related genes, including various types of cytokines, immunoreceptors, adhesion molecules and chemokines. Secretion of cytokines and inflammatory mediators plays important roles in uveitis. Studies have shown that the liver X receptor (LXR) stimulants supressed the inflammatory response and blocked the signal transduction of NF-κB signaling [26]. Some synthetic ligands, such as GW3965 and TO901317 (TO90), have shown greater efficacy and potency than the endogenous ligand of LXRs, the oxysterol. Studies on these ligands reveal some of the biological mechanisms in the LXR family. TO90 treatment reduced the levels of proinflammatory cytokines, including IFN-γ, TNF-α, IL-6, IL-1β, MCP-1 and IL-17, and decreased the expressions of the NF- κB subunit p65 at the mRNA and protein levels in a mouse model of experimental autoimmune uveitis (EAU) [27]. The role of Th17 cells in the pathogenesis of uveitis by secretion of inflammatory cyotkines i.e. IL-17 was summarized by Luger et al [28]. In addition, Th17 cells induce uveitis by directly targeting tissue cells. TO90 treatment also inhibited the expression of Th17 in EAU, which suggests that TO90 has the potential to become a novel therapeutic agent for autoimmune uveitis (Figure 5).

### NRs and retinopathy

#### NRs and age-related macular degeneration (AMD)

**Figure 6:**
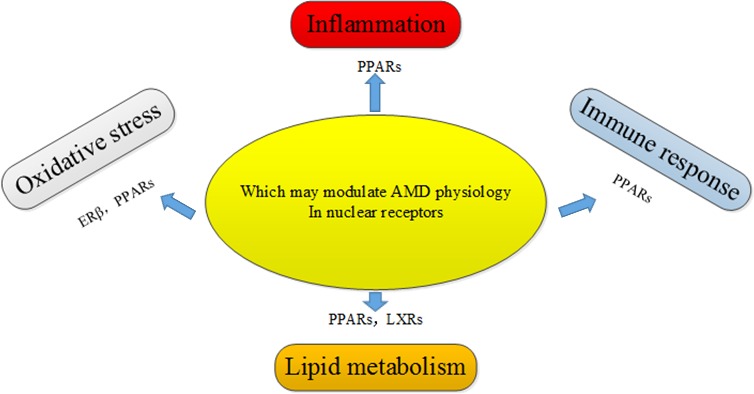
Potential nuclear receptors that may regulate AMD pathogenic pathways through their involvement in oxidative stress, inflammation, immune response, and lipid metabolism

AMD is a debilitating medical condition, accompanied by progressively impaired vision, and severely affects the living quality of elderly people. AMD is a major cause of blindness in the elderly in developed countries. Feskanich et al. [29] demonstrated that the decrease in endogenous levels of estrogen was associated with the risk of AMD in early postmenopausal women, and postmenopausal hormone therapy has a protective effect against the progression of AMD. A prospective study from the Netherlands showed that ERα are associated with the progression of AMD in older women [30]. Another study showed that 17β-Estradiol protects against oxidative stress-induced cell damage in ARPE-19 cells by targeting ERβ, and ERβ was found to be expressed in ARPE-19 cells, but not in the human RPE cells [31]. In addition, the association between PPAR/RXR signaling pathway and AMD has been gradually revealed, particularly since PPARγ was found to participate in the maintenance of lipid sensing, immunomodulation, and redox signaling, all of which were related to the pathogenesis of AMD. The isomers of PPAR/RXR have been found in human macrophages, RPE cells, and endothelial cells. Although the dysregulation of PPARγ has a limited effect on AMD, PPARγ may play an important role in modulating oxidative stress and inflammatory responses in AMD [32]. Though genetic studies have not found a direct association between heterodimers of LXRs/RXR and AMD, it is hypothesized that heterodimers of LXRs/RXR may function as complexed NRs in AMD. In addition, LXRs can reverse cholesterol transport to avoid the over-accumulation of lipids and thus maintain the balance between cholesterol and lipid acids, and these processes are dysregulated in AMD [33]. Sene et al. [34] found that intra vitreal delivery of LXR agonists restored the cholesterol efflux and alleviated the severe sides effect of photocoagulation-induced choroidal neovascularization (CNV) in old mice. Malek et al. [35] summarized several biological pathways that are potentially involved in the development and progression of AMD in great detail. Furthermore, the correlation between nuclear receptors and the disease was introduced in the same review. They believe that the role of nuclear receptors in the posterior segment of the eye is promising. One of the top priorities is to find the potential association of NR drug therapy with AMD (Figure 6).

#### NRs and pigmentary retinal degeneration (PRD)

NR2E3, also called photoreceptor-specific nuclear receptor (PNR), belongs to the eye organ receptor family with its expression restricted to the photoreceptors [36]. NR2E3 is postulated to maintain the survival, development, and differentiation of Rods by interacting with transcriptional factors, CRX and NRL [37]. NR2E3 protein expression is located in the outer layer of neurosensory retina. NR2E3 has a significant role in the development and function of rod cells [38]. *NR2E3* gene mutations resulted in the enhanced S-cone syndrome (ESCS) which is manifested by the excess of blue cones and a loss of rods, showing a gain of function of photoreceptors [39-41], Goldmann-Favre syndrome (GFS) [42], clumped pigmentary retinal degeneration (CPRD), retinitis pigmentosa (RP) et al. with common clinical features including night blindness and loss of functions of rods [43].

#### NRs and retinal neovascularization

**Figure 7:**
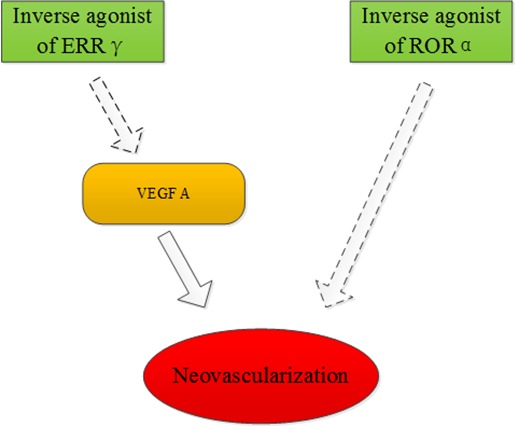
ERRγ and RORα may be targets for treatment of ischemic retinopathies

Retinoid acid receptor-related orphan receptor α (RORα) is a member of the orphan nuclear family and it has many biological functions including lipid metabolism and inflammatory modulation. The expression of RORα was substantially up-regulated in the oxygen-induced proliferative retinopathy mouse model. However, genetic deficiency of RORα significantly decreased pathological retinal neovascularization. It was shown that RORα directly suppressed the gene transcription of suppressors of cytokine signaling 3 (SOCS3), which is an important negative regulator of inflammation. SR1001, a RORα inverse agonist, can effectively protect against pathological neovascularization. These results demonstrate that RORα inhibition can be used for the treatment of ocular neovascularization [44]. It was found that hypoxic conditions (1% O2) and desferrioxamine treatment in a ganglion cell line (RGC-5) increased the mRNA and protein levels of ERRγ. This result indicated that ERRγ could be a target in the treatment of ischemic retinopathies [45]. Another study demonstrated that DNA-PK directly phosphorylates NOR-1 and, therefore modulates SMC proliferation, which contributes to the vascular remodeling processes [46] (Figure 7).

### NRs and ophthalmic tumors

**Figure 8:**
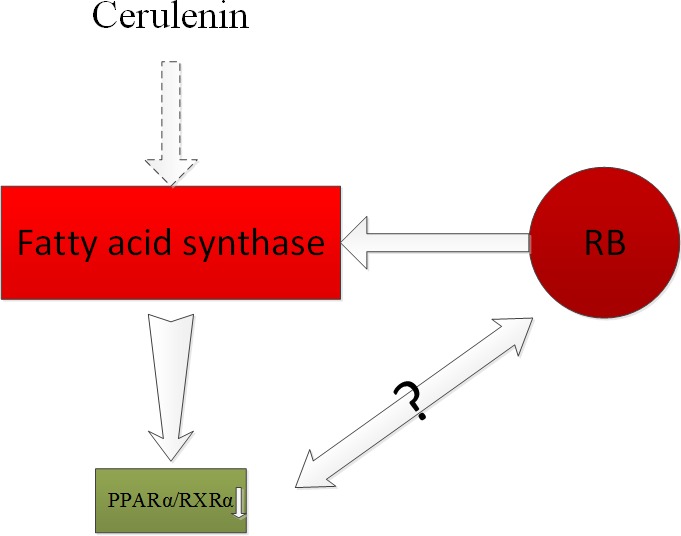
Fatty acid synthase (FASN) had a higher expression in retinoblastoma (RB) than that in normal cells, and was associated with high invasive ability The chemical inhibitor of FASN, cerulenin treatment resulted in altered expression levels of many genes, including the down-regulation of PPARα and RXRα in RB.

The question of whether ER and PR are expressed in conjunctival melanoma or uveal melanoma cells has attracted a number of investigations. In 1995, Foss et al. [47] proved that most of the conjunctival melanoma and uveal melanoma cells had expressed heat-shock protein 27 (HSP27), but the expression of ER and PR were not found in these melanoma cells. Therefore, Foss et al. concluded that ER and PR hardly play any roles in the progression of these melanomas. Studies from Grostern also found no histological evidence for the presence of type I estrogen receptors in choroidal melanoma [48]. Shown by a tissue-array, Pache [49]revealed the expression of PR, but not ER, in melanocytic lesions of the ocular conjunctiva, and thePR expression increased with age. The differences among these studies may be due to difference in human races from which tissues were extracted and the experimental methodologies employed. Fatty acid synthase (FASN) has a higher expression in retinoblastoma (RB) than that in normal cells, and is associated with high invasive ability. Studies also showed that treatment with the chemical inhibitor of FASN, cerulenin, resulted in altered expression levels of many genes, including down-regulation of PPARα and RXRα in RB [50]. The association between PPARα and RB may require further study. The functions of retinoic acid were studied by targeting its respective receptors on the ocular surface. It was shown that retinoic acid improved the partial and full thickness of corneal lacerations, as well as the defect of corneal epithelia [51]. Immunohistochemical staining with androgen receptor and adipophilin were used to separate squamous cell carcinomas, melanomas, sebaceous tumors, and basal cell carcinomas. Both squamous cell carcinomas and melanomas showed negative staining while basal cell carcinomas showed positive staining for androgen receptor in a distant minority of cells. For the intraepithelial (or pagetoid) spread, detection of androgen receptor was more sensitive and reliable than adipophilin [52]. Further study from Mulay et al. [53] concluded that AR is useful in diagnosing sebaceous carcinoma and differentiating it from SCC and BCC. Moreover, Kubota et al. [54], showed that the primary ductal adenocarcinoma of lacrimal gland specimens were positive for androgen receptor, and negative for PR and ER. Vaso-proliferative tumors of the retina (VPTRs) are a type of benign tumors that is common in the peripheral retina in the elderly. It can be spontaneous or secondary to other diseases of the eye. Its main characteristics in clinic are peripheral retinal vascular masses with obvious exudation [55]. Recent studies have found that a novel p.D406G mutation in the NR2E3 gene is associated with VPTRs [56]. This discovery is beneficial for disease diagnosis and would potentially provide another form of therapy for the affected patients (Figure 8).

## NR AND THERAPY

Endogenous or synthetic ligands that induce activation or inhibition of the respective receptors to regulate the expressions of targeting genes have been extensively used for medical treatment [57]. For instance, the selective AR antagonist, flutamide, has been used for the treatment of prostate cancer; tamoxifen, which partially antagonizes ER, has been used to treat breast cancer; troglitazone indirectly activates PPARγ to reduce blood glucose levels in patients with type II diabetes. It has been reported that a third-generation drug discovery effort has begun in the field of nuclear receptors for non-enzymatic therapeutic targets. It is believed that with the increased understanding of NRs and ocular diseases, more NR therapeutic drugs will be used in ophthalmology [58].

## SUMMARY

NRs play important regulatory roles in the development and progression of ocular diseases. The abnormal expression of NRs can lead to a variety of ocular pathogenesis. Continuous discovery of the relationship between nuclear receptors and ocular diseases not only has important significance in the study of the occurrence and development of diseases, but also brings inspiration for new drug development. It can make targeted therapy become a reality, and individualized medicine possible. At different stages of ocular diseases, there will be high expressions of respective NRs, and we can establish ocular disease animal models or *in vitro* cell experiments to investigate the expression of NRs at these stages, and subsequently develop respective agonists, inhibitors, co-activators, or co-repressors for the treatment of ocular diseases. In addition, the expressions of relevant NRs will be helpful for the diagnosis of ocular diseases. Therefore, it is of great scientific interest to put more effort in the study of NRs.

## Abbreviations

AF: Activation function
AMD: Age-related macular degeneration
AR: Androgen receptor
ATRA: All -trans retinoic acids
CNV: Choroidal neovascularization
COUP-TFs: Chicken ovalbumin upstream promoter-transcription factors
CPRD: Clumped pigmentary retinal degeneration
CVI: Cerebral visual impairment
DBD: DNA-binding domain
EAU: Experimental autoimmune uveitis
ER: Estrogen receptor
ER: Estrogen-related receptor
ESCS: Enhanced S Cone Syndrome
FASN: Fatty acid synthase
FK: Fungal keratitis
GR: Glucocorticoid receptor
HRE: Hormone response element
IL: Interleukin
LBD: Ligand-binding domain
LEC: Lens epithelial cell
LXR: Liver X receptor
MMPs: Matrix metalloproteinases
MR: Mineralcorticoid receptor
NLS: Nucleus localization sequence
NR: Nuclear receptor
PNR: Photoreceptor-specific nuclear receptor
PPAR: Peroxisome proliferator-activated receptor
PR: Progesterone receptor
RAR: Retinoic acid receptors
RB: Retinoblastoma
ROR: Retinoic acid receptor-related
RP: Retinitis pigmentosa
RPE: Retinal pigment epithelium
RXR: Retinoid X receptor
SOCS3: Suppressor of cytokinesignaling3
SR: Steroid hormone receptor
TR: Thyroid hormone receptor
VDR: Vitamin D3 receptor
VEGF: Vascular endothelial growth factor
